# Supplementary material for: HIVprotI: an integrated web based platform for prediction and design of HIV proteins inhibitors
Source: J Cheminform. 2018 Mar 9;10:12. doi: 10.1186/s13321-018-0266-y (PMC5845081; doi:10.1186/s13321-018-0266-y)
Supplement: Supplementary file 2 — Additional file 2. Source code of HIVProtI web server. [file 13321_2018_266_MOESM2_ESM.zip › HIVprotI_Source-code/hivprotI/weka-3-6-6/doc/weka/core/pmml/BuiltInArithmetic.html]

BuiltInArithmetic


---


|  |  |  |
| --- | --- | --- |
| **Overview** \| **Package** \| **Class** \| **Tree** \| **Deprecated** \| **Index** \| **Help** \| **Weka's home** | |  |
| PREV CLASS   **NEXT CLASS** | **FRAMES**    **NO FRAMES**     **All Classes** |  |
| SUMMARY: NESTED \| FIELD \| CONSTR \| METHOD | DETAIL: FIELD \| CONSTR \| METHOD |  |


---


## weka.core.pmml Class BuiltInArithmetic

```
java.lang.Object
  weka.core.pmml.Function
      weka.core.pmml.BuiltInArithmetic
```

**All Implemented Interfaces:**: java.io.Serializable

---

``` public class BuiltInArithmetic extends Function ```

Built-in function for +, -, \*, /.

**Version:**
:   $Revision 1.0 $

**Author:**
:   Mark Hall (mhall{[at]}pentaho{[dot]}com)

**See Also:**: Serialized Form

---

**Constructor Summary**

`BuiltInArithmetic(weka.core.pmml.BuiltInArithmetic.Operator op)`
  
          Construct a new Arithmetic built-in pmml function.


| **Method Summary** | |
| --- | --- |
| `Attribute` | `getOutputDef()`             Get the structure of the result produced by this function. |
| `java.lang.String[]` | `getParameterNames()`             Returns an array of the names of the parameters expected as input by this function |
| `double` | `getResult(double[] incoming)`             Get the result of applying this function. |
| `void` | `setParameterDefs(java.util.ArrayList<Attribute> paramDefs)`             Set the structure of the parameters that are expected as input by this function. |
| `java.lang.String` | `toString()` |
| `java.lang.String` | `toString(java.lang.String pad)` |

**Methods inherited from class weka.core.pmml.Function**

`getFunction, getFunction, getName`

**Methods inherited from class java.lang.Object**

`equals, getClass, hashCode, notify, notifyAll, wait, wait, wait`

**Constructor Detail**

### BuiltInArithmetic

```
public BuiltInArithmetic(weka.core.pmml.BuiltInArithmetic.Operator op)
```

:   Construct a new Arithmetic built-in pmml function.

    **Parameters:**: `op` - the operator to use.


**Method Detail**

### setParameterDefs

```
public void setParameterDefs(java.util.ArrayList<Attribute> paramDefs)
                      throws java.lang.Exception
```

:   Set the structure of the parameters that are expected as input by
    this function. This must be called before getOutputDef() is called.

    :   **Specified by:**: `setParameterDefs` in class `Function`
    :   **Parameters:**: `paramDefs` - the structure of the input parameters **Throws:**: `java.lang.Exception` - if the number or types of parameters are not acceptable by this function

---


### getParameterNames

```
public java.lang.String[] getParameterNames()
```

:   Returns an array of the names of the parameters expected
    as input by this function

    :   **Specified by:**: `getParameterNames` in class `Function`
    :   **Returns:**: an array of the parameter names

---


### getOutputDef

```
public Attribute getOutputDef()
```

:   Get the structure of the result produced by this function.
    Subclasses must implement.

    :   **Specified by:**: `getOutputDef` in class `Function`
    :   **Returns:**: the structure of the result produced by this function.

---


### getResult

```
public double getResult(double[] incoming)
                 throws java.lang.Exception
```

:   Get the result of applying this function.

    :   **Specified by:**: `getResult` in class `Function`
    :   **Parameters:**: `incoming` - the arguments to this function (supplied in order to match that of the parameter definitions **Returns:**: the result of applying this function. When the optype is categorical or ordinal, an index into the values of the output definition is returned. **Throws:**: `java.lang.Exception` - if there is a problem computing the result of this function

---


### toString

```
public java.lang.String toString()
```

:   **Overrides:**: `toString` in class `Function`

---


### toString

```
public java.lang.String toString(java.lang.String pad)
```

:   **Overrides:**: `toString` in class `Function`


---


|  |  |  |
| --- | --- | --- |
| **Overview** \| **Package** \| **Class** \| **Tree** \| **Deprecated** \| **Index** \| **Help** \| **Weka's home** | |  |
| PREV CLASS   **NEXT CLASS** | **FRAMES**    **NO FRAMES**     **All Classes** |  |
| SUMMARY: NESTED \| FIELD \| CONSTR \| METHOD | DETAIL: FIELD \| CONSTR \| METHOD |  |


---
